# An Expanded Interplay Network between NF-κB p65 (RelA) and E2F1 Transcription Factors: Roles in Physiology and Pathology

**DOI:** 10.3390/cancers14205047

**Published:** 2022-10-14

**Authors:** Spyros Foutadakis, Eugenia Roupakia, Panagiotis Liakopoulos, Petros Kolovos, Evangelos Kolettas

**Affiliations:** 1 Center of Basic Research, Biomedical Research Foundation, Academy of Athens, 115 27 Athens, Greece; 2 Laboratory of Biology, School of Medicine, Faculty of Health Sciences, and Institute of Biosciences, University Research Center of Ioannina (U.R.C.I.), University of Ioannina, University of Ioannina Campus, 451 10 Ioannina, Greece; 3 Institute of Biomedical Research (IBR), Foundation for Research and Technology (FORTH), University of Ioannina Campus, 451 10 Ioannina, Greece; 4 Department of Molecular Biology and Genetics, Democritus University of Thrace, 681 00 Alexandroupolis, Greece

**Keywords:** NF-κB, E2F1, genomics, transcription factor interactions/interplay, lung cancer

## Abstract

**Simple Summary:**

Members of the NF-kappaB (κB) and the E2F transcription factor (TF) families regulate the expression of numerous genes controlling several biological processes, including cell proliferation and viability, metabolic pathways, pro-inflammatory, immune and stress-like responses, maintaining cell homeostasis, and physiology. While the NF-κB RelA/p65 and E2F1 TFs functions are quite distinct, they regulate the transcription of several common genes, but their interplay in target-gene promoters can either activate, blunt, or repress gene expression in different cell types affecting several processes, with physiological consequences. Using available genomics data, we showed that the RelA/p65 TF subunit binds to distal active enhancers, while the E2F1 TF binds to gene promoters. Further, RelA/p65 may attract and recruit E2F1 in gene promoters, acting potentially as a recruiting TF to properly control the transcription of target genes, a potential mechanism allowing for concerted actions of RelA/p65 and E2F1 in response to mitogenic, inflammatory, or genotoxic stimuli.

**Abstract:**

Transcription Factors (TFs) are the main regulators of gene expression, controlling among others cell homeostasis, identity, and fate. TFs may either act synergistically or antagonistically on nearby regulatory elements and their interplay may activate or repress gene expression. The family of NF-κB TFs is among the most important TFs in the regulation of inflammation, immunity, and stress-like responses, while they also control cell growth and survival, and are involved in inflammatory diseases and cancer. The family of E2F TFs are major regulators of cell cycle progression in most cell types. Several studies have suggested the interplay between these two TFs in the regulation of numerous genes controlling several biological processes. In the present study, we compared the genomic binding landscape of NF-κB RelA/p65 subunit and E2F1 TFs, based on high throughput ChIP-seq and RNA-seq data in different cell types. We confirmed that RelA/p65 has a binding profile with a high preference for distal enhancers bearing active chromatin marks which is distinct to that of E2F1, which mostly generates promoter-specific binding. Moreover, the RelA/p65 subunit and E2F1 cistromes have limited overlap and tend to bind chromatin that is in an active state even prior to immunogenic stimulation. Finally, we found that a fraction of the E2F1 cistrome is recruited by NF-κΒ near pro-inflammatory genes following LPS stimulation in immune cell types.

## 1. Introduction

In eukaryotic cells, gene expression is tightly controlled to attain the required levels of gene products in specific cell types, at specific times, and in response to a variety of intracellular and extracellular stimuli and multiple changes in the environment. To achieve this, eukaryotic gene expression is regulated by several mechanisms and at different levels to produce a gene product, and these include regulation of chromatin remodeling and transcription [1,2,3,4,5,6,7].

Gene expression at the chromatin and transcriptional levels is controlled by a combination of trans-acting factors operating at their target genes [8,9]. This involves expression, activation, and binding of different transcription factors (TFs) in their respective regulatory regions in DNA. Many TFs bind to their cognate DNA sequences (consensus motifs) and recruit transcriptional cofactors in target gene promoters and enhancers and act cooperatively to regulate gene expression. Many TFs function as ‘‘master regulators’’ to control the expression of different genes and several cellular processes and specific pathways in different cell types and during development [2,5,9,10]. The same TFs can regulate different genes in different cell types by collaborating through different ways such as facilitating each other in binding DNA (cooperative binding) or by influencing the chromatin state or transcription through different mechanisms (synergistic regulation). TFs can directly recruit RNA polymerase and/or cofactors acting as ‘‘co-activators’’ or ‘‘co-repressors’’ such as chromatin modifying and remodeling factors to regulate transcription. TFs are characterised as either ‘‘activators’’ or ‘‘repressors’’ of transcription, but this depends on the recruitment of multiple cofactors and epigenetic regulators that have opposite effects in a cell-type and stimulus-dependent context [1,3,4,6,9].

Among TF families, the family of NF-κB TFs regulate pro-inflammatory and/or stress-like responses, acting mainly as transcriptional activators, but also as transcriptional repressors in a cell-, context- and stimulus-dependent manner [11,12,13,14,15,16,17,18,19].

NF-κB TFs bind to DNA as dimers. The dimers are formed between five subunits, RelA/p65, c-Rel, RelB, p50, and p52. The RelA/p65-p50 heterodimer, an archetypical dimer of the NF-κB TF family present in most cells, is held by IκBs (Inhibitors NF-κB), such as the prototype IκBα, in the cytoplasm of cells not experiencing stress-like or inflammatory responses. Activation of RelA/p65-p50 heterodimers occurs by a canonical NF-κB signalling pathway, which is mediated by an upstream IKKβ serine/threonine activating kinase leading to phosphorylation, ubiquitination and proteasomal degradation of IκBα, allowing the RelA/p65-p50 heterodimers to translocate to the nucleus and regulate the expression of their target genes [12,13,14,20].

The occurrence of TF binding sites in gene promoters and enhancers, as well as the relative occupancy of TFs on specific sites may dictate whether a gene is transcriptionally active or repressed [3,4,6,10,21,22,23]. Importantly, mutations in TFs and TF-binding sites underlie many human diseases [5,8,24].

Previous studies have provided evidence of the interaction of members of the NF-κB and E2F TF families in modulating transcription factor responses. The family of E2F TFs are essential for cell survival and proliferation. E2Fs are key regulators of cell cycle progression, and their transcriptional activities are regulated by histone acetyltransferases (HATs). Retinoblastoma (Rb) family proteins (pRb, p107, and p130) bind to E2Fs and inhibit their transcriptional activities by disrupting HAT binding and recruitment of histone deacetylases [25,26,27,28]. For example, E2F1 is recruited to chromatin to activate essential cyclin genes such as cyclin E [29] and Cdc6 [30,31] leading to S phase entry.

NF-κB [12,13,14,20] and E2F TFs [25,26,27,28,32] and their signalling pathways play important roles in cellular growth control and viability and are often subject to deregulation in cancer. However, NF-κB and E2F functions are quite distinct and can also antagonise each other. While NF-κBs generally promote cell proliferation and survival, E2Fs can act as either transcriptional activators or suppressors of cellular growth. Genes encoding proteins that control cell cycle progression (*E2F1*, *CCND1*, *CDC6*, *CDKN1A*/*p21*, CDKN1B/*p27*) and DNA damage response (DDR) (*ATM*, *GADD45β*) are differentially regulated by E2Fs and NF-κBs, yet the mechanisms allowing for concerted actions of NF-κBs and E2Fs in response to a wide range of stimuli such as mitogenic, inflammatory, and genotoxic stimuli remain poorly understood [15,16,33,34,35,36,37,38,39,40,41,42].

E2F1 suppresses canonical NF-κB activity thereby promoting apoptosis [33,34], and vice versa, NF-κB also suppresses E2F-target gene expression [15,16] suggesting that the functions of activating E2Fs and NF-κBs can be mutually antagonistic. IKKβ activation initially inhibited cell growth in short-term sub-culturing of human diploid fibroblasts (HDFs) [15] and murine embryonic fibroblasts (MEFs) [16] by suppressing E2Fs and their targets. In the latter study, it was shown that the activation of the IκB kinases, IKKα or IKKβ, inhibited cell growth and E2F-dependent transcription in normal HDFs. The inhibition of E2F by IKKs was not observed in cells lacking NF-κB RelA/p65, but it was observed in cells lacking the Rb family genes. RelA/p65 disrupted the physical interaction between activator E2Fs (E2F1-3) and the HAT cofactor transactivation/transformation-domain associated protein, resulting in a reduction in E2F-responsive gene expression. Furthermore, IKKα and IKKβ directly phosphorylated E2F4, resulting in nuclear accumulation and enhanced DNA binding of the E2F4/p130 repressor complex. This growth inhibitory system involves an Rb-independent suppression of E2Fs by the IKK/NF-κB signalling pathway. Thus, an NF-κB-dependent mechanism for growth arrest mediated by a dual mechanism. E2F-1-dependent transcription was inhibited by IKK activation and E2F-4 was phosphorylated directly by IKK resulting in increased activity of the E2F-4/p130 repressor complex [15].

Studies showed that E2F1 could physically associate with RelA and/or its major dimer partner p50 [34,43,44]. A reciprocal and coordinated control of transcription by E2F1 and NF-κB has been shown. Ectopic expression of E2F1 was shown to reduce canonical NF-κB transcriptional activity, and expression of RelA/p65 was also shown to reduce *CCNE* transcription [45]. It was also shown that activation of NF-κB in response to inflammatory cytokines depends on the cell cycle phase and the expression of E2F TFs. Specifically, it was shown that the NF-κB response was stronger in cells that were entering S-phase than in cells that were already in S-phase replicating their DNA. The activating E2F1, which accumulates in G_1_/S-phase was shown to interact with RelA to alter expression of certain genes. However, during S-phase, the repressing E2F4 binds to RelA and represses its activation. Thus, the interplay between NF-κB and E2F regulates the timing of inflammatory signalling and cell proliferation [45].

NF-κB and E2F1 TFs have been known to regulate the transcription of several common genes, while their interplay in target-gene promoters affects several processes in different cell types. The human E2F1 gene promoter contains potential E2F1 and NF-κB binding sites. E2F1 promoter activity is associated with NF-κB in quiescent cells and NF-κB is replaced by E2F1 in concert with gene activation early (at 12 h) after serum stimulation, later (by 24 h) Rb is recruited to E2F1-promoter complexes to counterbalance E2F1’s activity [38]. Studies have suggested that E2F1 and other TFs may be recruited by NF-κB to activate the promoters of selective target genes [44,45,46,47], suggesting that NF-κB recruitment alone is insufficient to induce the transcription of many genes without participating partner TFs. Antagonism between E2F1 and NF-κB impacts on *BNIP-3* gene transcription in apoptotic responses, wherein NF-κB may determine if E2F1 mediates proliferation or death [41]. In human cardiac AC16 cells, NF-κB and E2F1 antagonise for the pyruvate dehydrogenase kinase 4 (PDK4) promoter and in playing a role in the metabolic profile of these cells during inflammation [48]. A genome-wide study has also identified that upon TLR4 activation E2F1 is recruited by NF-κB to target-gene promoters [44]. However, the regulation of inflammatory response genes by NF-κB are more complex and binding of NF-κB to target promoters is also influenced by nucleosomal positioning [46,49,50,51,52,53,54].

Genomic analyses of NF-κB regulated transcriptome has suggested roles in the regulation of several enhancers during IL-1 stimulation [55], while in lymphoblastoid cells, in which both canonical and non-canonical pathways are active, a complex pattern has been documented, involving FOXM1 as a co-regulator of a large fraction of NF-κB genes [56]. The complexity of NF-κB binding patterns is also evidenced by the variation in the consensus NF-κB binding motif as well as the fact that about half of NF-κB bind to sites that lack any consensus NF-κB binding motifs [54,56]. In contrast, individual genomic analyses of E2F1 sites has revealed that E2F1 binds mostly in the vicinity of promoters of several genes that play several physiological roles, such as the regulation of cell proliferation and survival studies [57,58].

In the post-genomic era, the availability of sequence and occupancy data makes it possible to consider genome-wide interactions between TFs and their binding sites. In this study, we present a comparative and comprehensive study of the RelA/p65 and E2F1 cistromes in different cell types by integrating epigenomics and RNA-seq data.

## 2. Materials and Methods

For this study we re-analysed the following publicly available datasets: GSM1517085 (DNaseI-seq_DMSO), GSM1517089 (H3K27ac DMSO ChIP-seq), GSM1517093 (p300 DMSO ChIP-seq), GSM1517095 (p300 TNFα ChIP-seq) [51], GSM604656 (p65 DMSO WT ChIPseq), GSM604658 (p65_TNFα WT ChIP-seq) [59], GSM558469 (E2F1 HeLa ChIP-Seq) [60], GSM1693906 (E2F1 U2OS ChIP-seq) [61] GSM2975777 (p65 U2OS control ChIP-seq), GSM2975779 (p65 U2OS TNFα 4h) [62], GSM881056-59 (E2F1 mouse dendritic cells ChIP-seq), GSM881111-14 (p65 mouse dendritic cells ChIP-seq), GSM881080-83 (H3K27ac ChIP-seq mouse dendritic cells) [63], GSM3045697-102 (RNA-seq for control and E2F1 overexpression in U2OS cancer cells) [64], and GSM5354346-349 (RNA-seq for TNFα-treated and control HeLa cancer cells) [65].

Bioinformatics analyses were carried out with the Galaxy suite [66]. The quality of the sequencing reads was evaluated using the FastQC algorithm. To map sequencing reads, the Bowtie2 algorithm was used with the very sensitive end-to-end option and the hg19 version of the human or the mm9 version of the mouse genome [67]. Duplicates were discarded using the RmDup command from Samtools [68]. Peaks were called with the MACS2 algorithm using a q-value cutoff of 0.05 [69]. The bedtools suite was used to perform arithmetics between genomic intervals [70]. Bigwig files were prepared with the bamCoverage command from the Deeptools suite [71]. The IGV browser was used to produce individual snapshots [72].

Heat maps were constructed with the computeMatrix and plotHeatmap options from Deeptools. Upset plots were produced using the Upset Diagram option from within Galaxy [73].

Gene ontologies for the closest genes to peaks were found using the GREAT tool [74] with the single closest gene (1000 kb) option. The genomic distribution of peaks was derived using the CEAS software [75] from cistrome [76].

Motif analysis was carried out using the Meme-ChIP software [77]. 

For RNA-seq experiments, reads were mapped to hg19 version of the human genome using HISAT2 [78]. Reads falling into genes were calculated with HTSeq [79]. Differential gene expression analysis was done using DESeq2 [80].

## 3. Results

### 3.1. Genomic Landscape of p65 and E2F1 in Cancer Cells

**Figure 1:**
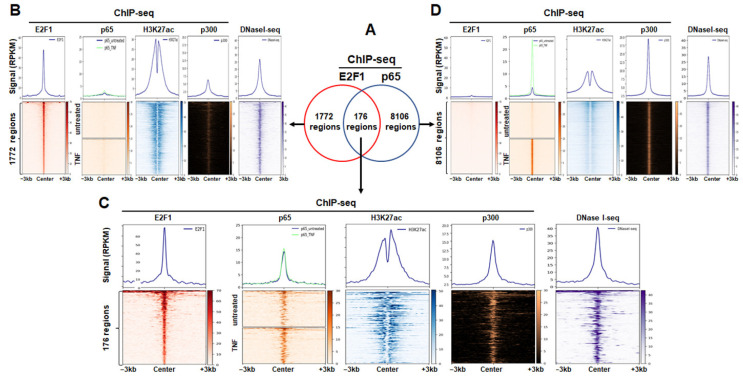
(**A**) Venn plots depicting the common E2F1-p65, the E2F1-specific, and the p65-specific binding regions (cistromes) in HeLa cancer cells. (**B**–**D**) Aggregate plots (**up**) and heatmaps (**down**) illustrating the signal distribution for E2F1, p65, H3K27ac, and DNaseI-seq accessibility around the E2F1-specific, the common E2F1-p65, and the p65-specific binding regions, respectively.

To study the genomic binding landscapes of p65 and E2F1 and their overlap in HeLa cancer cells, we re-analysed publicly available ChIP-seq data for p65 following TNFα stimulation [59], and E2F1 prior to TNFα stimulation [60]. We identified 1948 peaks for E2F1 and 8282 peaks for p65 in HeLa cancer cells, prior and upon TNFα stimulation, respectively. Interestingly, 176 regions of the E2F1 peaks are co-localised with p65 upon TNFα stimulation (Figure 1A, Supplementary Figure S1, Supplementary Table S1). 

Next, we studied the epigenomics landscape, the genomic distribution, the underlying motif profiles for TFs as well as gene ontologies for the closest genes to shared and transcription factor specific peaks. Focusing on E2F1-specific peaks we found that they mainly reside in an accessible chromatin environment bearing active histone marks as revealed by intersecting peaks with H3K27ac, p300, and DNaseI-seq (Figure 1B) [51]. The same holds true for p65-specific peaks and the common E2F1-p65 peaks (Figure 1C,D).

### 3.2. Genomic Distribution of p65 and E2F1 Peaks

**Figure 2:**
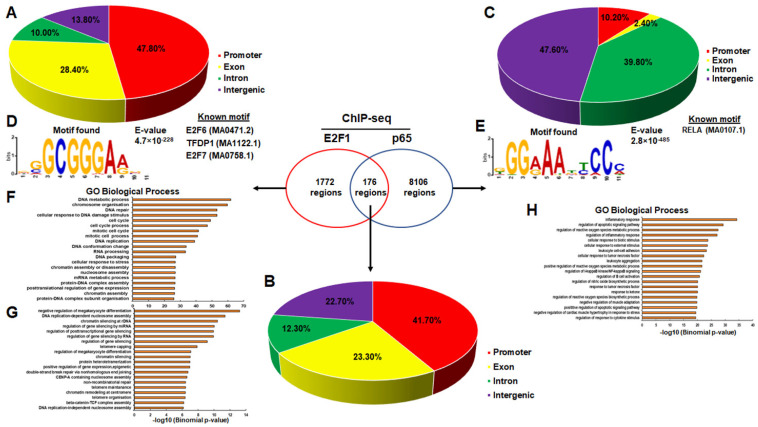
(**A**–**C**) The figure panels depict the genomic distribution of E2F1-specific, E2F1-p65 common, and p65-specific peaks respectively in HeLa cells. (**D**,**E**) The figure panels show the top motif for E2F1-specific and p65-specific peaks, respectively. (**F**–**H**) The figure panels illustrate the top biological processes for E2F1-specific, E2F1-p65 common, and p65-specific peaks, respectively.

Next, we analysed the genomic distribution of E2F1-specific peaks and found an extremely high preference for promoters (47.8%), while 28.4% were exonic, 13.8% intergenic, and only 10% were intronic (Figure 2A). This high preference for promoter sequences is characteristic of E2F members and has previously been described [81]. An analogous distribution was found for common E2F1-p65 peaks with 41.7% falling in promoters, 23.3% in exons, 22.7% in intergenic regions, and 12.3% in intronic regions (Figure 2B). On the other hand, the genomic distribution of p65-specific peaks was more balanced with 10.2% in promoters, 2.4% in exons, 47.6% in intergenic regions and 39.8% in intronic regions (Figure 2C). De novo motif analysis for the E2F1-specific peaks recovered motifs of the E2F family as the most statistically significant as expected (Figure 2D), RELA was the most significant motif for p65-specific peaks (Figure 2E), while no significant motifs were recovered for E2F1-p65 peaks due to the relatively small number of peaks. Gene ontology analysis using the GREAT algorithm for the closest genes to E2F1-specific peaks (Supplementary Table S2) recovered among others significantly enriched processes related to cell cycle and DNA repair (Figure 2F) in accordance with the well-described role of E2F factors in the above processes [82]. For the common E2F-p65 peaks, gene ontology analysis identified processes related to gene silencing by miRNA and DNA replication-dependent nucleosome assembly to be significantly enriched (Figure 2G), while for p65-specific peaks processes related to inflammatory responses and immune processes were the most significant (Figure 2H) in accordance with the well-established role of p65 in the above processes [83].

**Figure 3:**
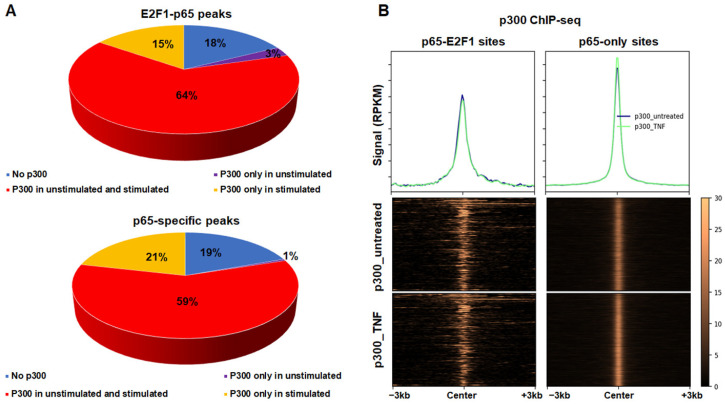
(**A**) Shown are E2F1-p65 common (**up**) and p65-specific peaks categorised according to whether they bear p300 binding prior and upon TNFα treatment in HeLa cancer cells. (**B**) Shown is the p300 signal around the common p65-E2F1 and p65-only peaks prior and upon TNFα treatment.

To identify whether p65 binds to chromatin that carries active chromatin marks even prior to stimulation, we re-analysed p300 ChIP-seq data prior and upon TNFα stimulation [51]. We found that the majority of E2F1-p65 common peaks (64%) and p65-specific peaks (59%) reside in chromatin that is bound by p300 prior and upon stimulation (Figure 3). Only a small proportion of the E2F1-p65 common peaks (18%) and p65-specific peaks (19%) are attracting p300 upon TNFα stimulation. Finally, 15% of the E2F1-p65 common peaks and 21% of the p65-specific peaks are without p300 both prior and upon TNFα stimulation. These highlight the preference of p65 and E2F1 to positively regulate their target genes.

### 3.3. Transcriptional Profile of the p65 and E2F1 Targeted Genes

**Figure 4:**
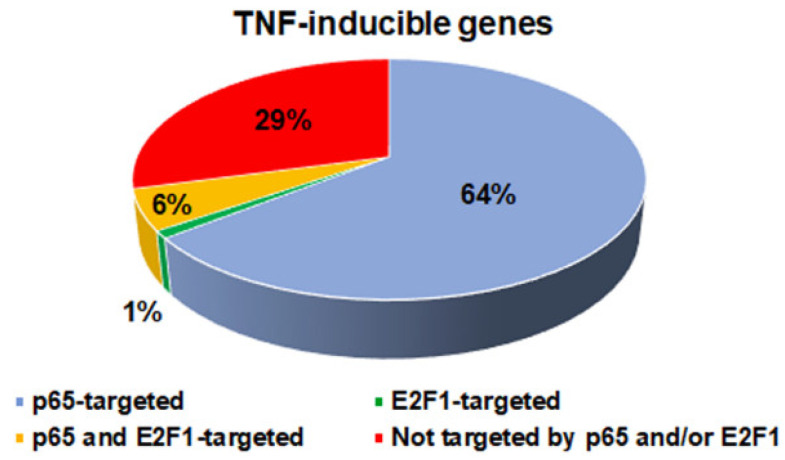
The percentage of TNFα-inducible genes in HeLa cells targeted by p65 alone or by E2F1-p65.

In order to identify the effect of p65 and E2F1 in the transcriptomics profile of their target genes, we intersected the aforementioned p65 and E2F1 peaks with publicly available RNA-seq data [65] from control and LPS-stimulated HeLa cells. We found that from a total of 202 upregulated genes following TNFα treatment (log2fold change > 0.5; FDR < 0.05), 130 (64%) are targeted and thus putatively regulated only by p65 (Supplementary Table S3), underscoring the well-known pivotal role of p65 in the regulation of the anti-inflammatory responses. On the other hand, there are only two (1%) genes targeted exclusively by E2F1. Moreover, we have identified 11 (6%) genes targeted by E2F1 and p65, consisting of 1 gene targeted exclusively by p65-E2F1 complex and 10 genes targeted by p65 and E2F1 peaks which are however not co-localised (Figure 4). The above findings could indicate that E2F1 is not directly involved in the pre-establishment of the anti-inflammatory programme at least in resting cells. Regarding the 10 downregulated genes following TNFα treatment, 5 were found to host p65-only TNFα inducible peaks in their vicinity, while no E2F1-specific or common E2F1-p65 peaks were found in their premises.

### 3.4. p65 and E2F1 Cistromes in Different Human Cell Types

To examine if the E2F1 and p65 cistromes are shared between cell types and whether they exhibit universal characteristics we analysed E2F1 [61] and p65 [62] ChIP-seq datasets from U2OS cancer cells. We identified 1664 p65 binding sites following TNFα stimulation and 450 E2F1 binding sites of which 89 are co-bound by the two factors (Supplementary Figure S2A). A large fraction of the above binding sites resides in regions decorated by H3K27ac (Supplementary Figure S2B–D). 

In accordance with the experiments in HeLa cells, E2F1 binding sites show a high preference for promoter regions (Supplementary Figure S3A,B), are highly enriched for E2F family motifs (Supplementary Figure S3D) and are found in the vicinity of genes involved in processes related to cell cycle and DNA repair (Supplementary Figure S3F,G). As expected, binding sites for p65 display a typical genomic distribution (Supplementary Figure S3C) and are highly enriched for the p65 motif (Supplementary Figure S3E).

**Figure 5:**
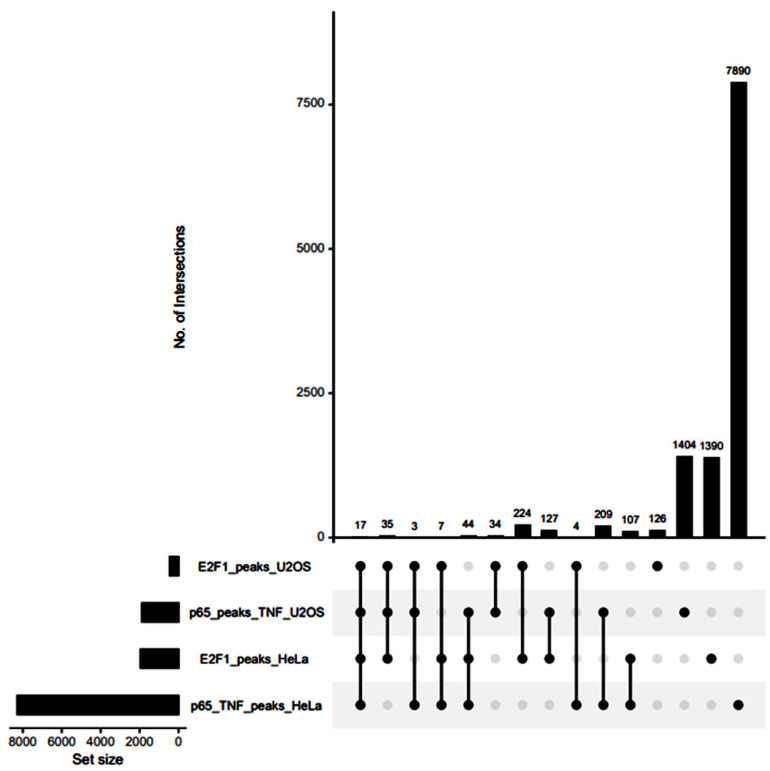
Upset plot showing multi-way comparisons between p65 and E2F1 binding sites in HeLa and U2OS cells.

By comparing the E2F1-specific cistromes in HeLa and U2OS cells, we discovered a high overlap with 224 peaks in common, while 209 p65 peaks were shared between the two cell types (Figure 5). Moreover, we could identify only 17 E2F1-p65 common binding sites that are shared between HeLa and U2OS cells (Supplementary Table S1). Taken together with data shown above, we can conclude that the genomic overlap of these two TFs is rather limited and not highly conserved between different cell types.

In order to identify genes regulated by E2F1, we integrated them with RNA-seq data in U2OS cells following overexpression of E2F1 [64]. We identified 1077 genes upregulated following E2F1 overexpression and 527 downregulated genes (log2-fold change > 1, padj. < 0.05). By integrating RNA-seq with E2F1 ChIP-seq experiments, we were able to identify 24 genes which are upregulated following E2F1 overexpression and have E2F1 binding sites in their vicinity. These genes are provided in Supplementary Table S4. The above could indicate that not all E2F1 binding have regulatory potential and lead to functional outcomes as previously suggested for other factors [84].

### 3.5. p65 and E2F1 Cistromes in Mouse Cells

In order to investigate the dynamics of the binding pattern of both E2F1 and p65, following treatment with an immunogenic stimulus such as TNF or LPS, we focused on E2F1 and p65 ChIP-seq data following LPS stimulation in mouse dendritic cells [63]. This would allow to investigate if there is a change in the co-localisation of the two factors following stimulation and the formulation of a model regarding the recruiting order and mechanism for the assembly of these stimulus-specific cistromes. Unfortunately, to the best of our knowledge, there are no genome-wide datasets describing the binding pattern of E2F1 following an immunogenic stimulation in human cells. Available ChIP-qPCR data in human monocytes following stimulation with LPS showed recruitment of E2F1 to selected inflammatory gene promoters in a mechanism that is dependent on NF-κB activation [44].

**Figure 6:**
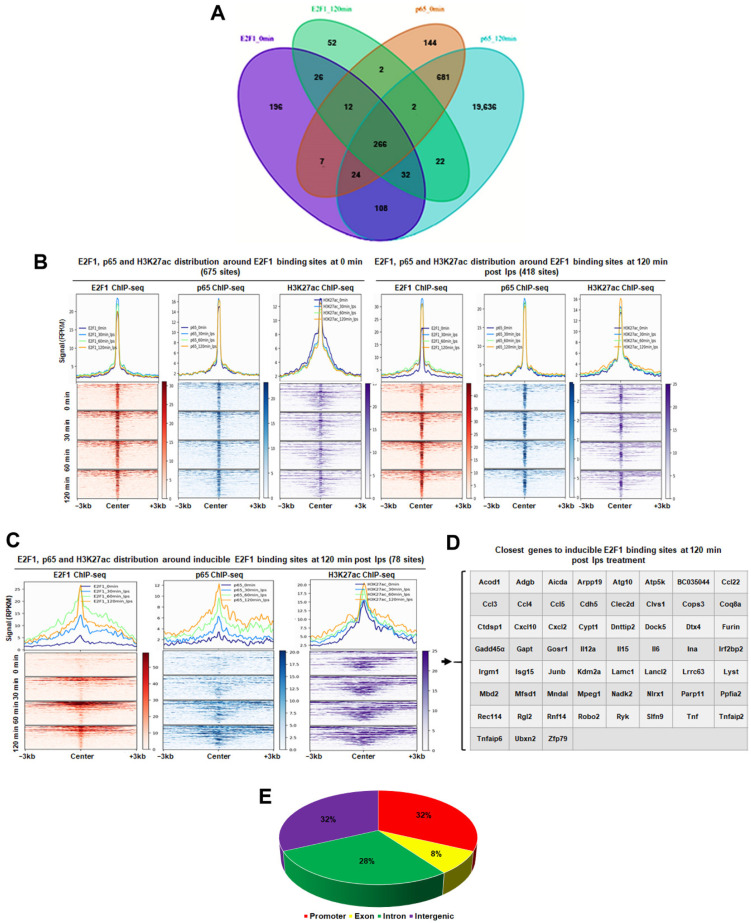
(**A**) Venn diagram depicting comparisons between E2F1 and p65 peaks prior and upon treatment with LPS for 120 min. (**B**) Aggregate plots (**up**) and heatmaps (**down**) depicting E2F1, p65, and H3K27ac levels around the E2F1 peaks at basal state (**left**) and 120 min after LPS treatment (**right**). (**C**) Aggregate plots (**up**) and heatmaps (**down)** showing E2F1, p65, and HK327ac levels around the 78 inducible E2F1 peaks 120 min after LPS treatment. (**D**) Closest genes to E2F1 inducible peaks 120 min after LPS treatment. (**E**) The genomic distribution of E2F1 inducible peaks 120 min after LPS treatment.

Peak calling analysis identified 675 E2F1 binding sites and 1147 p65 sites in unstimulated cells of which 310 are shared between the two factors (Figure 6A illustrates multiway comparisons; Supplementary Table S5). Following LPS stimulation for 120 min, 418 E2F1 peaks were found and 20,780 p65 binding sites of which 325 where shared (Figure 6A illustrates multiway comparisons).

We next turned our attention to the 78 newly acquired E2F1 binding sites that were only found following LPS stimulation (Figure 6C). We found that p65 is recruited to several of these sites with similar kinetics for the two factors. Moreover, most of these sites reside in an active chromatin environment as they are decorated by the activating histone modification H3K27ac even at the basal state (Figure 6C). Most importantly, many of the E2F1 inducible sites are found in the vicinity of key inflammatory genes such as *CCL5*, *TNF-A*, *IL6,* etc. (Figure 6D) with a genomic distribution heavily biased towards promoters (Figure 6E). Apart from promoters, sites with inducible E2F1 and p65 binding were equally distributed between intergenic and intronic elements. Taken together with the results from Lim et al., 2007 [44], these data point towards a conserved stimulus provoked co-localisation of E2F1 and p65 near pro-inflammatory genes important for the correct execution of the pro-inflammatory programme at least in immune cells.

## 4. Discussion

The families of NF-κB [12,13,14,20] and E2F [26,27,28] TFs serve several physiological functions and also contribute to several pathological states such as inflammatory diseases and cancer. In the current study, we selected the most known members of each family, namely RelA/p65 and E2F1, since numerous studies suggest an interplay between the two TFs in many diverse biological processes [15,16,45,48]. These processes include the regulation of pro-inflammatory and DNA damage responses, cell proliferation, cell survival, autophagy, and cell metabolism [33,34,45,48]. However, to our knowledge, no study has ever provided a detailed, comparative analysis of the genomic binding landscapes of these two factors by analysing multiple types of genomics data. 

To gain an understanding on the genome-wide binding capacity of both E2F1 and RelA/p65 TFs, we analysed and integrated data from ChIP-seq experiments in HeLa and U2OS cells. Based on our analysis in human cells, the genome-wide binding patterns and binding capacity is considerably different for the two TFs. A large fraction of RelA/p65 binding sites reside in intergenic regions and introns, in a large distance from the transcription start sites (TSS) (Figure 2C). This binding pattern suggests an enhancer-specific binding, in gene poor regions [47,85]. This notion is also supported by the fact that a fraction of NF-κB binding sites are known to bind within Alu transposable elements [86]. In contrast, the E2F1 binding sites reside mostly upstream of genes, in 5′ UTRs and exons, near TSS (Figure 2A). The binding pattern of E2F1 suggests a promoter specific, high-affinity binding, similar to the pattern found by previous studies [57,58]. Moreover, the NF-κB RelA subunit and E2F1 cistromes have a rather limited overlap in human cells and tend to bind chromatin that is in an active state even prior to immunogenic stimulation (Figure 3B and Figure 6B). The scarcity of available genome-wide binding data for E2F1 following treatment with an immunogenic stimulus in human cells precluded direct testing of the co-localisation of E2F1 and p65 under stimulatory conditions. Thus, we proceeded to the analysis of the only available E2F1 genome-wide binding dataset following LPS stimulation in mouse dendritic cells [63] and identified the stimulus provoked assembly of a common E2F1 and p65 cistrome in the vicinity of key pro-inflammatory genes. Similar conclusions were previously derived using ChIP-qPCR for E2F1 and selected promoters of pro-inflammatory genes in human monocytes following LPS [44]. In the latter study, p65 activation was necessary for the LPS-stimulated recruitment of E2F1 to chromatin and E2F1 was indispensable for the correct execution of the pro-inflammatory gene programme [44]. Taken together the above results indicate the existence of a conserved stimulus-provoked assembly of a common E2F1-p65 cistrome necessary for the accurate spatiotemporal execution of the pro-inflammatory gene programme at least in certain immune cell types.

Genomic regions bound by p65 or E2F1 are often characterised as potential activating elements [52,53,54,87]. Interestingly, E2F1 regions are mainly located around promoter regions, whereas p65 specific regions (Figure 2 and Supplementary Figure S3) are primarily located in intergenic or intragenic genomic regions. Collectively and based on previous findings [54,88], we deduce that before TNFα (or LPS) stimulation a large fraction of genomic regions already host the necessary “signatures” for active regulatory elements (H3K27ac, p300, DNaseI-seq accessibility), which subsequently are going to be bound by p65 upon TNFα (or LPS) stimulation (Figure 1, Figure 3 and Figure 6). Based on the modus operandi of p65 derived from previous studies [54,88], these active regulatory elements could pre-loop to their target genes before TNFα stimulation, followed by the recruitment of p65 on these regions upon TNFα stimulation, leading to the activation or repression of the target genes depending on which TFs co-localise with p65 or even the type (canonical or non-canonical) DNA binding motif of p65 [54,88].

**Figure 7:**
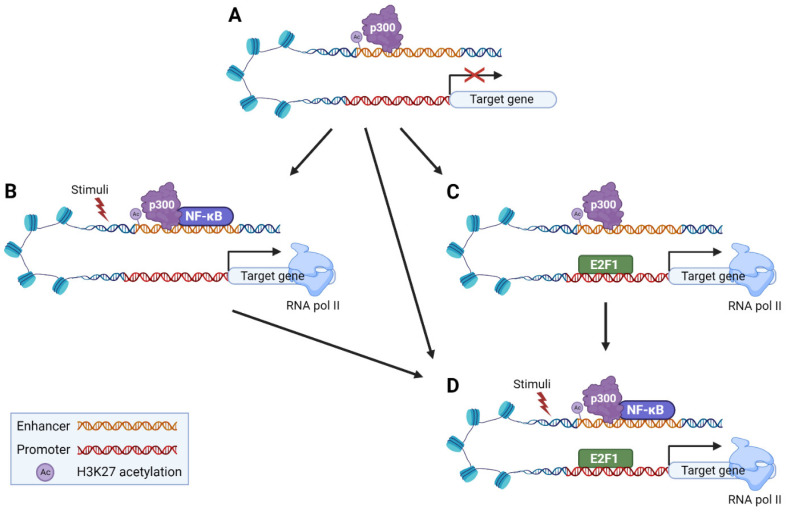
Model for the proper transcriptional regulation of the E2F1-p65 target genes. (**A**) Prior to stimulation, intergenic or intragenic active regulatory elements, which have all the necessary “signatures” for active regulatory elements, are looped to the promoter of the target genes, prior to stimulation. (**B**) Upon stimulation, the loop is preserved, p65 is recruited to the active regulatory elements activating its target genes. At the same time, for the p65-E2F1 target genes, (**C**) E2F1 could bind first and (**D**) then p65 is recruited. Alternatively, (**B**) p65 which is already bound on chromatin, (**D**) can attract and recruit E2F1 on chromatin. Otherwise, (**D**) E2F1 and p65 bind chromatin independently of each other (Please see text for more details).

Thus, it is tempting to speculate that for the proper transcriptional regulation of the E2F1-p65 target genes, intergenic or intragenic active regulatory elements, which have all the necessary “signatures” for active regulatory elements, are looped to the promoter of the target genes, prior to stimulation (Figure 7A). Upon stimulation, the loop is preserved, p65 is recruited to the active regulatory elements activating its target genes (Figure 7B). At the same time, E2F1 could bind on chromatin independently of p65 (Figure 7C) regulating the E2F1 target genes and then p65 is recruited on chromatin to regulate the p65-E2F1 target genes (Figure 7D). Alternatively, p65 can bind first on chromatin (Figure 7B) and then attract and recruit E2F1 on chromatin (Figure 7D). Alternatively, E2F1 and p65 can bind chromatin independently of each other (from Figure 7A directly to Figure 7D). Thus, p65 may act potentially as a recruiting TF forming an active chromatin hub, in order for the TFs to be in close proximity in the 3D nuclear space to form a complex leading to the proper transcriptional control of the target genes.

## 5. Conclusions

Overall, p65 and E2F1 are important TFs, which appear to work in a synergistic manner to regulate their target genes. Our model provides a potential mechanism allowing for concerted actions of RelA/p65 and E2F1 TFs in response to mitogenic, inflammatory, or genotoxic stimuli.

## Data Availability

Publicly available datasets were analysed in this study. These data can be found here: GSM1517085 (DNaseI-seq_DMSO), GSM1517089 (H3K27ac DMSO ChIP-seq), GSM1517093 (p300 DMSO ChIP-seq), GSM1517095 (p300 TNFα ChIP-seq) [51], GSM604656 (p65 DMSO WT ChIPseq), GSM604658 (p65_TNFα WT ChIP-seq) [59], GSM558469 (E2F1 HeLa ChIP-Seq) [60], GSM1693906 (E2F1 U2OS ChIP-seq) [61], GSM2975777 (p65 U2OS control ChIP-seq), GSM2975779 (p65 U2OS TNFalpha 4h) [62], GSM881056-59 (E2F1 mouse dendritic cells ChIP-seq), GSM881111-14 (p65 mouse dendritic cells ChIP-seq), GSM881080-83 (H3K27ac ChIP-seq mouse dendritic cells) [63], GSM3045697-102 (RNA-seq for control and E2F1 overexpression in U2OS) [64], and GSM5354346-349 (RNA-seq for TNFα-treated and control HeLa) [65].

## Supplementary Materials

The following supporting information can be downloaded at: https://www.mdpi.com/article/10.3390/cancers14205047/s1, Figure S1: IGV screenshots (A) p65-specific peaks at the NFKBIA locus. (B) E2F1-specific and HeLa-specific peak at the CCNA2 locus. (C) p65-E2F1 peak at the Stat1 locus. (D) p65-E2F1 HeLa specific peak at the OAS3 locus. (E) p65, E2F1 peaks at the Myc locus; Figure S2: (A) Venn plots depicting the common E2F1-p65, the E2F1-specific and the p65-specific binding regions in U2OS cells. (B–D) Aggregate plots (up) and heatmaps (down) illustrating the signal distribution for E2F1, p65 and H3K27ac around the E2F1-specific, the common E2F1-p65 and the p65-specific binding regions respectively; Figure S3: (A–C) Figure panels depict the genomic distribution of E2F1-specific, E2F1-p65 common and p65-specific peaks respectively in U2OS cells. (D,E) show the top motif for E2F1-specific and p65-specific peaks, respectively. (F–H) Figure panels illustrate the top biological processes for E2F1-specific, E2F1-p65 common and p65-specific peaks respectively; Table S1: Provides binding sites for p65 and E2F1 in HeLa and U2OS; Table S2: Provides the closest genes to p65 and E2F1 binding sites in HeLa and U2OS; Table S3: Provides TNFα-inducible genes in the vicinity of p65 and E2F1 binding sites in HeLa; Table S4: Provides a list of upregulated and downregulated genes following E2F1 overexpression in U2OS cells that have E2F1 binding in their vicinity; Table S5: List of binding sites for E2F1 and p65 prior and upon treatment with LPS for 120 min in mouse dendritic cells.
