# Supplementary material for: An Expanded Interplay Network between NF-κB p65 (RelA) and E2F1 Transcription Factors: Roles in Physiology and Pathology
Source: Cancers (Basel). 2022 Oct 14;14(20):5047. doi: 10.3390/cancers14205047 (PMC9600032; doi:10.3390/cancers14205047)

Figure S1: IGV screenshots (A) p65-specific peaks at the NFKBIA locus. (B) E2F1-specific and HeLa-specific peak at the CCNA2 locus. (C) p65-E2F1 peak at the Stat1 locus. (D) p65-E2F1 HeLa specific peak at the OAS3 locus. (E) p65, E2F1 peaks at the Myc locus

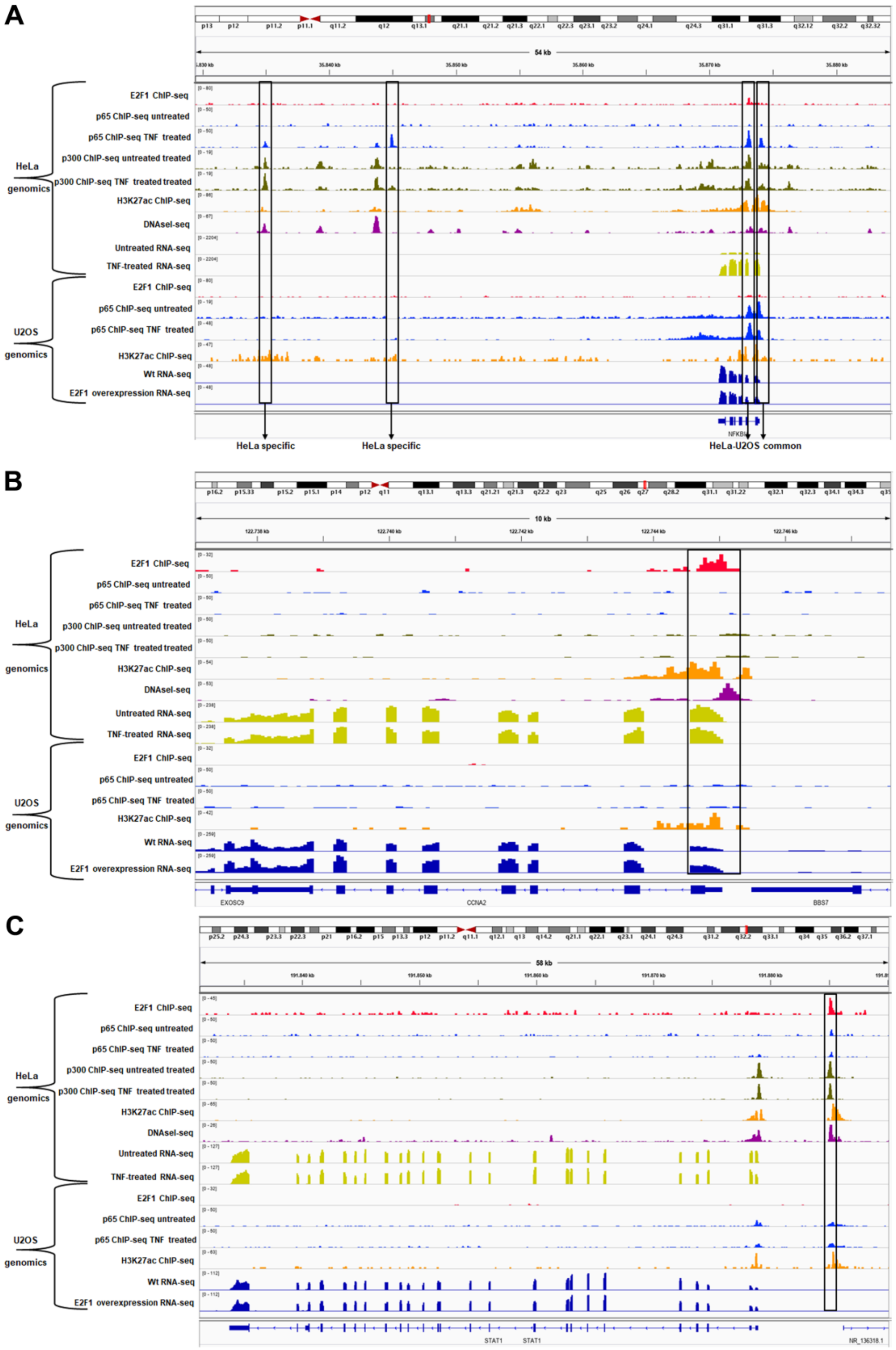

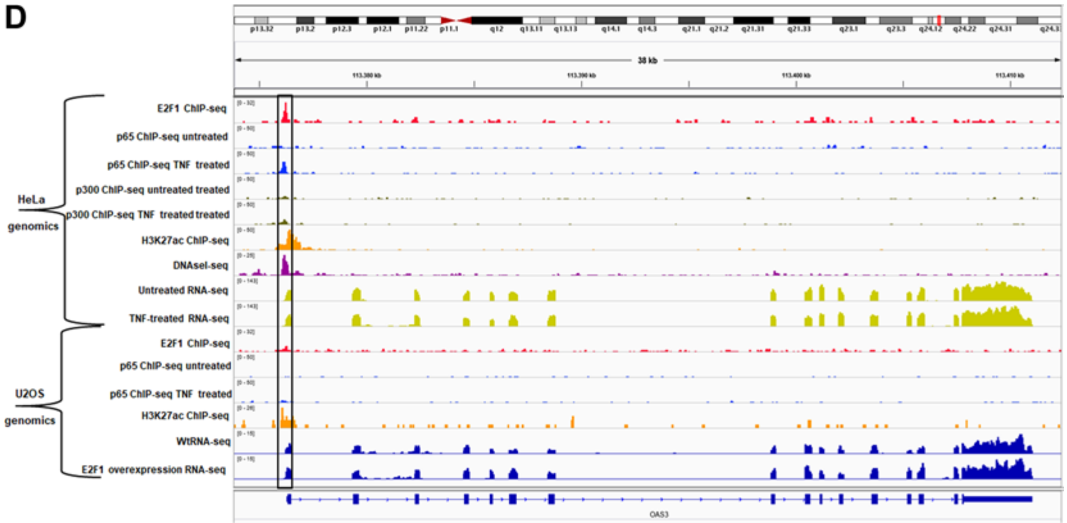
D
HeLa genomics
E2F1 ChIP-seq
p65 ChIP-seq untreated
p65 ChIP-seq TNF treated
p300 ChIP-seq untreated treated
p300 ChIP-seq TNF treated treated
H3K27ac ChIP-seq
DNAseI-seq
Untreated RNA-seq
TNF-treated RNA-seq
U2OS genomics
E2F1 ChIP-seq
p65 ChIP-seq untreated
p65 ChIP-seq TNF treated
H3K27ac ChIP-seq
WtRNA-seq
E2F1 overexpression RNA-seq
38 kb
OAS3

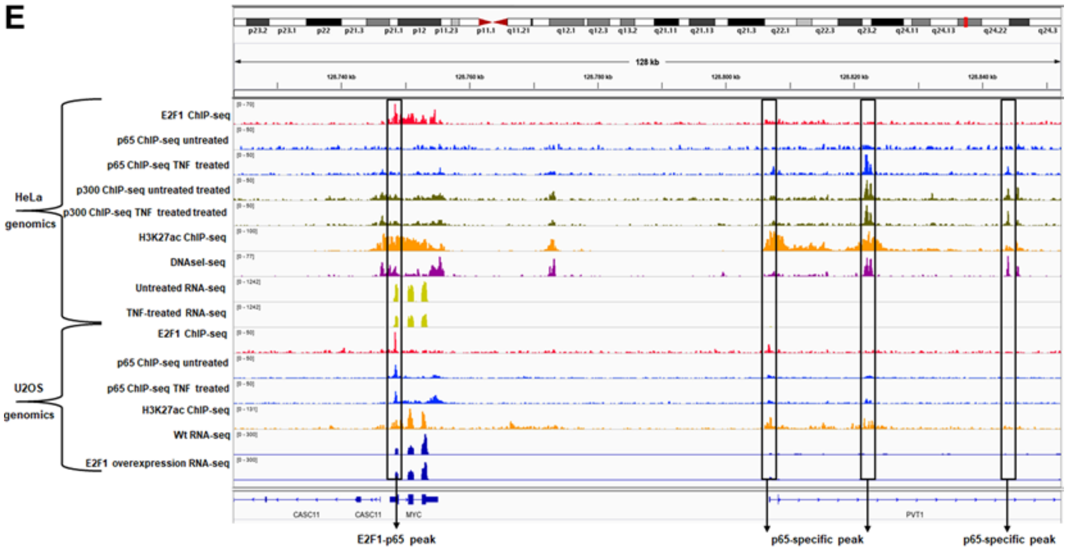
E
HeLa genomics
E2F1 ChIP-seq
p65 ChIP-seq untreated
p65 ChIP-seq TNF treated
p300 ChIP-seq untreated treated
p300 ChIP-seq TNF treated treated
H3K27ac ChIP-seq
DNAseI-seq
Untreated RNA-seq
TNF-treated RNA-seq
U2OS genomics
E2F1 ChIP-seq
p65 ChIP-seq untreated
p65 ChIP-seq TNF treated
H3K27ac ChIP-seq
Wt RNA-seq
E2F1 overexpression RNA-seq
128 kb
CASC11
CASC11
MYC
PVT1
E2F1-p65 peak
p65-specific peak
p65-specific peak

Figure S2: (A) Venn plots depicting the common E2F1-p65, the E2F1-specific and the p65-specific binding regions in U2OS cells. (B, C, D) Aggregate plots (up) and heatmaps (down) illustrating the signal distribution for E2F1, p65 and H3K27ac around the E2F1-specific, the common E2F1-p65 and the p65-specific binding regions respectively

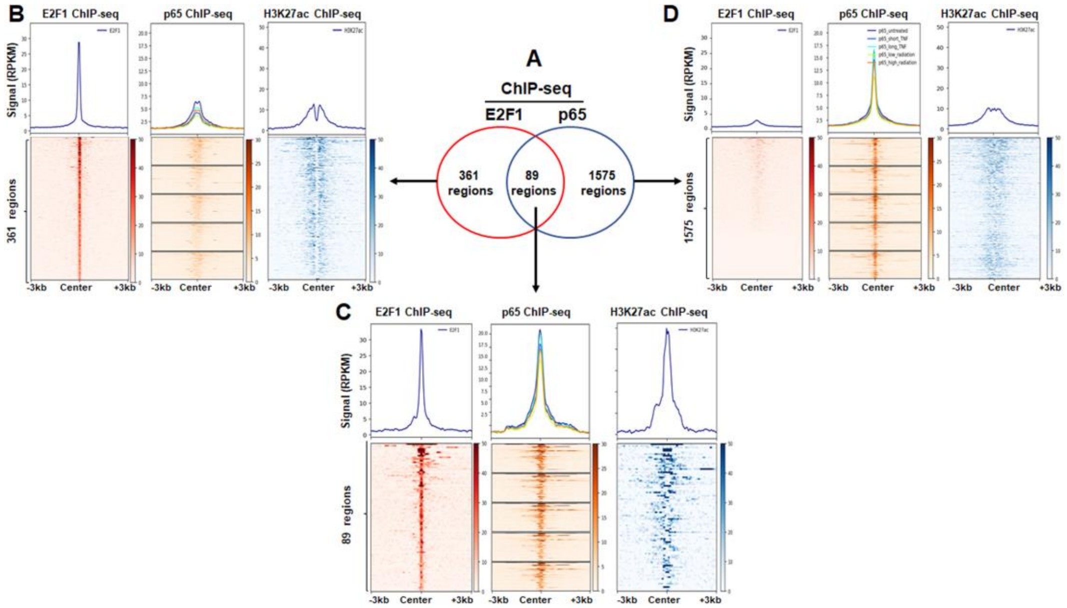

Figure S3: (A, B, C) Figure panels depict the genomic distribution of E2F1-specific, E2F1-p65 common and p65-specific peaks respectively in U2OS cells. D and E show the top motif for E2F1-specific and p65-specific peaks respectively. (F, G, H) Figure panels illustrate the top biological processes for E2F1-specific, E2F1-p65 common and p65-specific peaks respectively

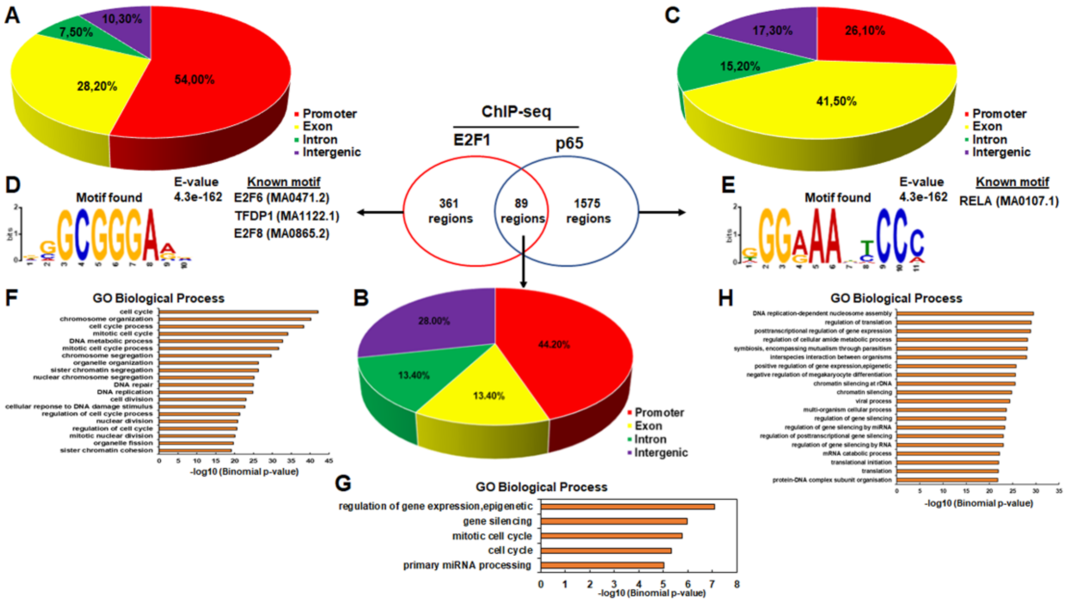

Supplement: Supplementary file 1 [file cancers-14-05047-s001.zip › Supplementary figures.pdf]
